# GTpick: A deep neural network for Cryo-EM particle detection

**DOI:** 10.1016/j.csbj.2025.10.029

**Published:** 2025-10-16

**Authors:** Shenhuan Ni, Chenghui Yang, Yutao Liu, Yuncong Zhang, Yiyan Shi, Anthony Qian, Ren Kong, Shan Chang

**Affiliations:** a Institute of Bioinformatics and Medical Engineering, School of Electrical and Information Engineering, Jiangsu University of Technology, Changzhou, Jiangsu 213001, China; b School of Pharmacy, Nanjing University of Chinese Medicine, Nanjing 210023, China; c Stevenson School, Pebble Beach, CA 93953, USA

**Keywords:** Cryo-EM, Particle detection, CryoSPARC, 3D structure reconstruction

## Abstract

Accurate identification of protein particles in cryo-electron microscopy (Cryo-EM) images is crucial for achieving high-resolution three-dimensional (3D) structural reconstruction. However, this task faces multiple challenges, including low signal-to-noise ratios, densely distributed particles, and class imbalance. To address these issues, this study proposes a target detection algorithm named GTpick, built upon the DETR framework. GTpick introduces a cross-attention mechanism to enhance the interaction between target queries and specific image features. In addition, a grouped one-to-many label assignment strategy is employed to improve recall in densely populated regions, and a Focal Loss function is incorporated to mitigate the adverse effects of background noise and class imbalance on detection accuracy. Experiments on large-scale Cryo-EM datasets demonstrate that GTpick outperforms existing machine learning-based particle-picking methods in terms of the resolution of 3D density maps reconstructed from detected particles and achieves superior Recall and F1 scores, particularly excelling in the Recall metric.

## Highlights

• GTpick is a Cryo-EM particle detection model built upon the DETR framework.
• GTpick achieves higher recall and true positive counts, demonstrating strong capability in Cryo-EM particle detection.
• Detection results from GTpick lead to improved 3D reconstruction quality when processed with CryoSPARC.
• GTpick still shows room for improvement in performance when applied to very large datasets.

## Graphical Abstract

**Figure:**
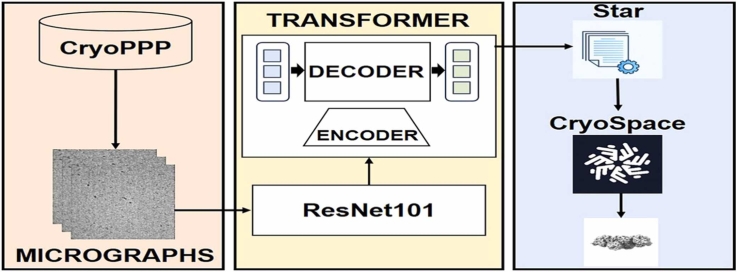

## 1 Introduction

Cryo-electron microscopy is an important technique for studying the three-dimensional structures of biological macromolecules such as proteins. In recent years, it has made significant progress in resolving difficult-to-crystallize samples, including membrane proteins and complex protein assemblies. Cryo-EM overcomes the stringent requirements of crystal preparation required for conventional X-ray crystallography by rapidly cryo-fixing biological samples in a close-to-physiological state. Its standard analytical processes include detection, alignment, classification, averaging, inverse projection of target particles, and ultimately 3D reconstruction of the protein structure [1]. Given this workflow, the quality of particle detection is a major factor that directly determines the accuracy and resolution of the final, reconstructed structure.

However, current particle detection methods still face significant challenges, especially for images with low signal-to-noise ratios, dense particle distributions, and/or severe levels of background interference. Traditional computer vision methods struggle to extract high-level features effectively and have limited detection capability [2], [3], [4]. Existing approaches can be categorized into two types: template matching and machine learning. Template matching relies on predefined reference templates to compare with candidate image regions, but this approach is limited if particle morphology varies greatly or when the background is complex; moreover, this procedure is highly susceptible to ice contamination and background noise.

Machine learning-based methods have substantially improved the automation of particle detection, although important limitations persist. For example, the APPLE picker [5] trains classifiers using positive and negative samples, but it has only been validated on a limited dataset, and its generalization performance remains uncertain. DeepPicker [6] adopts the VGG-Net [7] structure to frame the detection task as image classification; however, its use of sliding-window cropping limits efficiency. FastParticlePicker [8] uses a Fast R-CNN framework to enhance detection speed but remains sensitive to background noise. DeepEM [9], which uses the AlexNet architecture [10] for iterative training, suffers from instability when data are limited. crYOLO [11] leverages the YOLO [12] target detection framework to achieve high-precision automated detection but lacks generalization when faced with significant variation in particle size and shape. DeepConsensus [13] aggregates the results of multiple methods to build a high-quality dataset, albeit with high computational complexity. Topaz [14] uses a positive-unlabeled learning framework requiring only a small number of labeled samples, but under default settings, it tends to select too many overlapping particles and false positives, often requiring manual threshold adjustment.

In recent years, the Transformer framework has been introduced into Cryo-EM particle detection. CryoTransformer [15] applies self-attention mechanisms to capture long-range dependencies between particles, compensating for the limitations of traditional convolutional networks in global feature extraction. However, its robustness is insufficient in noisy images, and its one-to-one label matching strategy limits performance in densely populated regions.

To address these challenges, this paper proposes an improved Transformer-based detection algorithm named GTpick. First, a cross-attention mechanism is added to the Transformer decoder to enhance spatial interaction between target queries and image features. Second, a grouped one-to-many label assignment strategy [16] is introduced to improve recall in densely populated regions. Third, Focal Loss [17] is used for classification to reduce the negative effects of class imbalance between background and target categories, helping the model focus more on difficult-to-recognize particles. Experimental results show that, compared with existing methods, GTpick significantly improves particle detection performance while maintaining a lightweight architecture. Moreover, 3D reconstruction using CryoSPARC software verifies that the particle coordinates generated by GTpick lead to higher resolution reconstructed structures.

## 2 Materials and methods

**Fig. 1:**
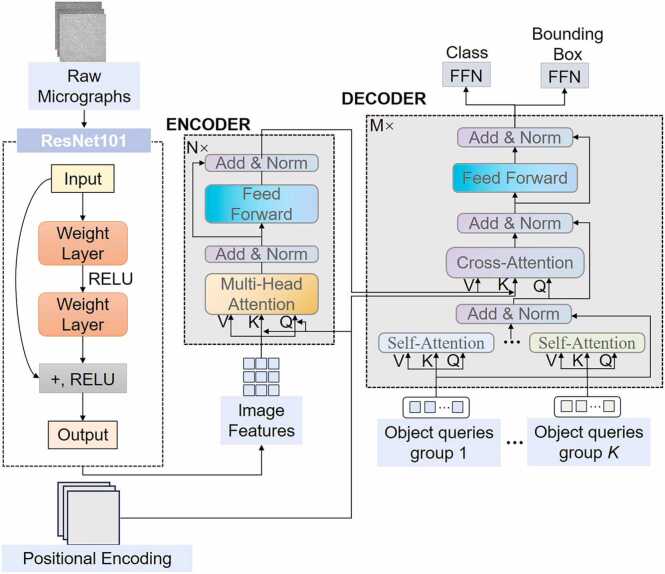
Overall architecture of the GTpick model. This model first uses ResNet101 to extract image features, then applies context modeling through a transformer encoder. The decoder uses self-attention and cross-attention to output class predictions and bounding box coordinates.

This study develops an end-to-end object detection network based on the DETR framework [18]. This approach consists of three primary modules: (1) a feature extraction module, (2) a transformer-based feature modeling module, and (3) a label matching and loss calculation module. The overall network architecture is illustrated in Fig. 1 To address the challenges posed by densely distributed targets and the difficulty of distinguishing targets from background in Cryo-EM particle detection tasks, this study introduces a series of customized improvements to the traditional Transformer decoder. Specifically, we incorporated a dynamic reference point mechanism to enhance positional awareness, as well as adaptive positional encoding [19] and cross-attention mechanisms to strengthen spatial information representation. In addition, a group-wise one-to-many assignment method is implemented in the label matching process, enabling more accurate detection of densely packed targets by improving recall. In the loss function design, the classification branch adopts Focal Loss to mitigate the impact of class imbalance, while the bounding box regression branch combined L1 Loss and GIoU Loss [20] to enhance localization accuracy.

### 2.1 Backbone network

In cryo-electron microscopy (Cryo-EM) images, target structures often exhibit blurred boundaries, if the background contains complex noise. Therefore, the backbone network must possess strong representational capabilities and robust feature extraction. To meet these requirements, this study adopts ResNet-101 as the primary feature extraction module and incorporates several structural optimization strategies to better integrate with the subsequent Transformer encoder.

We constructed the backbone network using a standard ResNet-101 architecture [21], we initialize the backbone network with pretrained weights, which accelerates model convergence under limited training samples and prevents the loss of details in Cryo-EM images. In addition, to address the instability of feature distribution caused by BatchNorm under small-batch training conditions [22], this study replaces standard BatchNorm with FrozenBatchNorm2d to ensure consistent feature distribution across various training settings.

### 2.2 Transformer module

When used to detect protein particles, Cryo-EM images typically suffer from low signal-to-noise ratios, blurred target boundaries, and issues related to the dense packing of individual proteins. Traditional convolutional neural networks (CNNs) are limited by their local receptive fields and can therefore struggle to model long-range context. These limitations manifest as false positives and missed detection opportunities. To address this problem, we introduce an improved Transformer architecture that performs global context modeling and target feature interactions. Our overall design follows the DETR framework and consists of an encoder and a decoder.

The original feature map output from the backbone network is denoted as $X\in R^{B\times C\times H\times W}$. To input this map into the Transformer encoder, it must first be flattened into a sequence format as follows: $F^{\left(0\right)}\in R^{\mathrm{HW}\times B\times C}$ where HW=H×W. To enhance the model’s perception of spatial positional information, we introduce a position encoding $P\in R^{H\times W\times C}$ based on trigonometric functions. This positional encoding is then added to the input feature map, which then forms the final input of the Transformer encoder: $Z^{\left(0\right)}=F^{\left(0\right)}+P$.

The primary function of the encoder module is to perform high-level contextual modeling on the feature set extracted by the backbone network, thereby enhancing the contextual understanding of the model. The encoder module itself consists of multi-head attention and feed-forward networks, accompanied by residual connections and layer normalization.

The decoder is composed of N stacked layers, each consisting of three sub-modules: self-attention, cross-attention, and a feed-forward network. The input to the decoder is the query vector q generated by the previous layer. Initially, the query vector is linearly projected to generate the query and key vectors, which are used to capture the relationships between different target instances. This is as follows: $q^{\prime }=qW_{q}$*,* $k^{\prime }=qW_{k}$ and its output is processed using residual connections and layer normalization:

(1) $$\begin{array}{c} q_{1}=\mathrm{LayerNorm}\left(q+\text{SelfAttention}\left(q^{\prime },k^{\prime },q\right)\right)\# \end{array}$$

The core innovation of the decoder is to compute cross-attention between the query vectors and the encoder output features as $M\in R^{\mathrm{HW}\times B\times C}$, while simultaneously introducing an explicit reference point positional encoding vector $P_{\mathrm{ref}}$ to enhance spatial localization. The resulting cross-attention is calculated as follows:

(2) $$\begin{array}{c} \text{CrossAttention}\left(q_{1}+P_{\mathrm{ref}},M,M\right)\# \end{array}$$

This output is then further processed through residual connections and layer normalization by:

(3) $$\begin{array}{c} q_{2}=\mathrm{LayerNorm}\left(q_{1}+\text{CrossAttention}\right)\# \end{array}$$

**Fig. 2:**
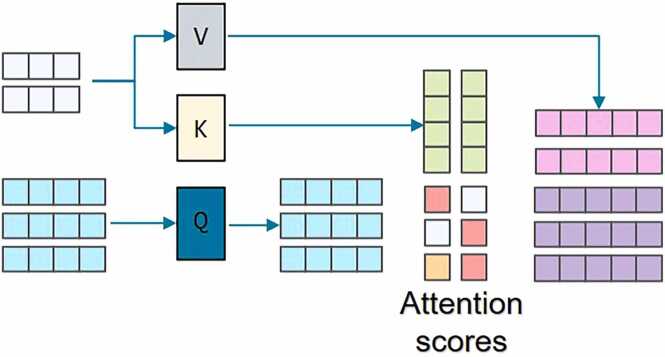
Structure of Cross-Attention Model. Here attention scores are computed as dot product operations between Q and K. They are then normalized and applied to V to produce the weighted output representation.

A conceptual diagram of the cross-attention module is shown in Fig. 2 By integrating content features with positional features, the cross-attention mechanism effectively enhances the model’s ability to distinguish between background and densely packed targets.

In standard DETR, the decoder employs a fixed number of query vectors, and each target instance can be matched to at most one query (i.e., “one-to-one matching”). In densely populated scenes, however, this may be insufficient and may lead to false negatives. To overcome this limitation, we introduce a group-wise query mechanism (group-wise matching), which explicitly partitions the query vectors into G sub-groups, each containing $Q_{g}$ sub-queries, where: $Q=G\times Q_{g}$.

During training, each group query independently participates in cross-attention calculations and interacts with target labels. For example, the cross-attention calculation process for the g group query is defined by: $q_{g}^{\prime }=q_{g}W_{q},k_{g}^{\prime }=MW_{k}$.

(4) $$\begin{array}{c} {\text{CrossAttention}}_{g}=\mathrm{Attention}\left(q_{g}^{\prime }+P_{g,\mathrm{ref}},k_{g}^{\prime },M\right)\# \end{array}$$

The output of the group query is then concatenated across calculation stages and combined for label matching and supervision optimization using the Hungarian Matching algorithm [23]. In general, this group-wise mechanism is designed to effectively enhance the detection of small particles and densely populated regions, and is useful for increasing local target coverage and enhancing the detection of protein particles captured in Cryo-EM images.

### 2.3 Loss function design and optimization

To facilitate end-to-end particle detection training using Cryo-EM images, this study designed a composite objective function that comprehensively considers target classification, bounding box regression, and overlap quality as three separate error components.

Next, we addressed the severe class imbalance problem in Cryo-EM images. To do so, we adopted Focal Loss [17] as the primary classification loss function; this was formalized as follows:

(5) $$\begin{array}{c} L_{\mathrm{cls}}=-\sum_{i=1}^{N}\alpha_{t}{\left(1-p_{t}\right)}^{\gamma }\log\left(p_{t}\right)\# \end{array}$$

Here, $p_{t}$ represents the predicted probability of the *i* target belonging to the correct class, $\alpha_{t}\in (0,1)$ is the class balancing factor. This loss effectively reduces the impact of easily classified negative samples, and thereby enhances the model’s focus on challenging classes.

For the bounding box regression, an L1 loss is employed to measure the positional difference between the predicted and ground truth coordinates:

(6) $$\begin{array}{c} L_{L1}=\left(\frac{1}{N}\right)\sum_{i=1}^{N}\left|{\hat{b}}_{i}-b_{i}\right|\# \end{array}$$

Here, ${\hat{b}}_{i}$ represents the center coordinates and scale (normalized) of the predicted bounding box, while $b_{i}$ denotes the corresponding ground truth box. Here we adopt GIoU Loss as a measure of the alignment between the predicted and ground truth boxes. Minimizing this value can effectively mitigate potential optimization difficulties caused by incomplete overlap. The GIoU Loss is defined as follows:

(7) $$\begin{array}{c} L_{\mathrm{GIoU}}=\left(\frac{1}{N}\right)\sum_{i=1}^{N}\left(1-\mathrm{GIoU}\left({\hat{b}}_{i},b_{i}\right)\right)\# \end{array}$$

Here, GIoU measures the overlap between the predicted and ground truth boxes which accounting for the minimum bounding region that encompasses both; the result is a more stable optimization target than can be obtained by calculating the traditional IoU (Intersection over Union) [12].

Finally, the total loss function is defined as a weighted sum of these three components:

(8) $$\begin{array}{c} L_{\mathrm{total}}=\lambda_{\mathrm{cls}}L_{\mathrm{cls}}+\lambda_{\mathrm{Ll}}L_{\mathrm{Ll}}+\lambda_{\mathrm{GIoU}}L_{\mathrm{GIoU}}\# \end{array}$$

This design achieves a unified optimization of target classification and localization quality, ensuring the model’s stable and high-precision performance in detecting densely packed small particles.

### 2.4 Post-processing and three-dimensional reconstruction

A.star file [24] is generated after completing training of the GTpick model. This is a standard format for storing metadata in Cryo-EM. In general, this file records key parameters for each particle found in the micrographs, including coordinates, rotation angles, and automated scoring. These data can be directly imported into CryoSPARC [25] to facilitate subsequent analyses of protein structure.

To do so we first import all Cryo-EM micrographs into CryoSPARC. Next, Contrast Transfer Function (CTF) estimation is performed to assess image quality and apply aberration correction. For example, CTF correction compensates for physical distortions that occur during imaging by enhancing the signal-to-noise ratio. This provides more accurate inputs for subsequent particle extraction and structure reconstruction steps.

Next, CryoSPARC extracts particles based on coordinate information found in the.star file, records the total number of particles, and automatically selects high-quality particles for further analyses. Following particle extraction, individual particle images are cropped from the micrographs, forming an initial particle dataset. This dataset then undergoes two-dimensional (2D) classification to improve data quality; this step removes abnormal or blurry particles and reduces noise interference in structure analysis. After 2D classification, high-quality particles with consistent morphology are selected for three-dimensional (3D) initial reconstruction; this process generates a low-resolution density map. The resolution of the reconstructed structure is then progressively enhanced via homogeneous refinement, heterogeneous refinement, and further local refinement steps.

Next, we calculated the Gold Standard Fourier Shell Correlation (GSFSC) to evaluate the reliability of the final reconstructed structure. This provides a quantitative measure of the resolution of the density map. GSFSC effectively assesses the structural signal within the reconstructed map, offering a confidence evaluation of the results.

Finally, we imported the optimized 3D density map into visualization software (e.g., UCSF Chimera [26]) to generate a clear and accurate three-dimensional protein structure model. This model can then be used to support subsequent structural analyses and studies of biological function.

## 3 Dataset and training setup and performance evaluation

### 3.1 Dataset

In this study, the CryoPPP [27], [28] public dataset is used for model training and evaluation. It is characterized by its large sample size, diverse particle types, and high-quality annotations. To construct the training process, the data are divided into training, validation, and test sets in a ratio of 8:1:1, and the ground truth particle coordinates provided in the dataset are used as supervisory signals to ensure annotation accuracy and consistency. All labeled information is uniformly organized in COCO format, with target category, bounding box location, and image attributes recorded in structured JSON files. This approach standardizes the data processing workflow, improves compatibility with mainstream detection frameworks, and provides a uniform and reliable foundation for model training and evaluation [15].

### 3.2 Experimental configuration and model training

**Fig. 3:**
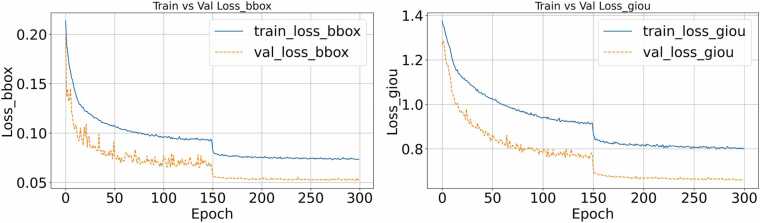
Bounding Box Loss and GIoU Loss curves of the model during training. In both the bounding box and GIoU panels, the loss values decrease continuously and converge after approximately 150 training epochs, indicating that the model has entered a stable optimization phase.

The experimental environment in this study is based on a CENTOS system, equipped with an NVIDIA RTX 4090 graphics card (24 GB of video memory). Model construction and training are implemented using the PyTorch 1.7.1 framework and CUDA 10.2. The training parameters are set as follows: the input size is 640 × 640; the AdamW optimizer [29] is used; the initial learning rate is set to 10⁻⁴; the learning rate of the backbone network is set to 10⁻⁵; and the weight decay parameter is set to 10⁻⁴. All network weights are randomly initialized using the Xavier initialization method [30]. In the attention mechanism and feed-forward network, a dropout of 0.1 is added before LayerNorm to enhance the model’s generalization ability. During training, the batch size is set to 2. Each training round takes approximately 10 min, and the total training time is 63 h. Fig. 3 shows the change curve of Bounding Box Loss and GIoU Loss during training.

As shown in Fig. 3, the overall trend of the two loss values is steadily decreasing, indicating that the model continues to achieve effective optimization in the target localization task. With the introduction of a regularization mechanism, the loss values decrease significantly and converge after approximately 150 rounds, indicating that the model has entered a stable training phase and is adequately learning target box positions.

### 3.3 Evaluation metrics

In evaluating particle selection algorithms in cryo-electron microscopy (Cryo-EM) images, two methods are commonly used.

The first method uses manually selected particles as a reference benchmark and employs precision, recall, and F1-score as evaluation metrics.

Another method involves importing particles selected by different algorithms into CryoSPARC software for 3D reconstruction. It then involves comparing the final resolution of the resulting 3D density maps. Using this approach, higher resolutions indicate better particle quality, more accurate localization, and finer structural reconstruction, thus indirectly reflecting the overall quality of the detection algorithm.

Using these two evaluation approaches, we next systematically compared and analyzed the performance of various Cryo-EM particle detection algorithms to verify the effectiveness and advantages of the proposed GTpick method regarding particle detection and 3D reconstruction.

## 4 Method validation and performance evaluation

### 4.1 Ablation experiments

To evaluate the contribution of each improvement proposed in this study to the performance of Cryo-EM particle detection, ablation experiments were performed on the EMPIAR-10017 dataset. This dataset, which contains β-galactosidase micrographs with moderate image size, a sufficient number of micrographs, and well-defined particle features, provides an ideal balance between computational tractability and biological complexity. These properties make EMPIAR-10017 a representative and efficient benchmark for evaluating model behavior under realistic conditions.

Four configurations were compared under identical training settings to ensure fairness and comparability. As shown in Table 1, Improvement 1, which integrates Cross-Attention and One-to-Many label assignment, achieved a Precision of 0.621, Recall of 0.434, and F1 Score of 0.511. Improvement 2, which replaces the loss function with Focal Loss while retaining Cross-Attention, alleviated class imbalance and background noise, yielding 0.618 Precision, 0.443 Recall, and 0.516 F1 Score. Improvement 3 combined One-to-Many assignment with Focal Loss, resulting in 0.596 Precision, 0.448 Recall, and 0.511 F1 Score. Improvement 4, which integrates all three strategies (Cross-Attention, One-to-Many, and Focal Loss), achieved the best performance (Precision = 0.643, Recall = 0.450, F1 = 0.529). These results demonstrate that the combined use of these modules synergistically enhances the accuracy and robustness of true particle detection.

**Table 1:** Results of Ablation study.

Model	Cross-Attention	One-to-Many	Focal Loss	Precision	Recall	F1 Score
Improvement 1	√	√	×	0.621	0.434	0.511
Improvement 2	√	×	√	0.618	0.443	0.516
Improvement 3	×	√	√	0.596	0.448	0.511
Improvement 4	√	√	√	**0.643**	**0.450**	**0.529**

### 4.2 Comparison experimental

#### 4.2.1 Comparative evaluation across multiple Cryo-EM datasets

In this study, we selected six representative cryo-electron microscopy (Cryo-EM) datasets, namely EMPIAR-10081, EMPIAR-11056, EMPIAR-10345, EMPIAR-10532, EMPIAR-10093, and EMPIAR-10947, all of which were obtained from the CryoPPP database. These datasets were used to systematically evaluate the performance of GTpick and other mainstream particle detection algorithms in terms of precision, recall, and F1-score. During the evaluation, the particle coordinates detected by each algorithm were matched against the manually annotated ground truth provided in the CryoPPP dataset to assess the coverage capability of each method in identifying protein particles in Cryo-EM images. Detailed information on the selected datasets is provided in Table 2.

**Table 2:** Detailed information of the five representative Cryo-EM datasets used for algorithm evaluation.

EMPAIR ID	Protein Type	Size (TB)	Image size	Particle Diameter (px)	Total Structure Weight (kDa)
10081	Transport Protein	0.052	(3710, 3838)	154	298.57
10093	Membrane Protein	0.097	(3838, 3710)	172	779.4
10532	Viral Protein	0.196	(4096, 4096)	174	191.76
11056	Transport Protein	0.164	(5760, 4092)	164	88.94
10345	Signaling Protein	0.085	(3838, 3710)	149	244.68
10947	Viral Protein	0.048	(4096, 4096)	240	443.92

To ensure a fair comparison, the CryoTransformer model was retrained on the same CryoPPP dataset using identical training parameters, preprocessing procedures, and hyperparameter settings as GTpick. In addition, both Topaz and crYOLO were fine-tuned (instead of directly using the official pretrained models) to better adapt to the CryoPPP dataset. The particle coordinates detected by each algorithm were subsequently used for downstream 2D classification and 3D reconstruction. Table 3 presents a systematic comparison of the detection performance of GTpick, CryoTransformer, Topaz, and crYOLO across the six EMPIAR test datasets.

**Table 3:** Comparison of particle detection performance of different methods on five EMPIAR datasets.

EMPIAR ID	Particles In ground Truth	Recall				Precision					F1 Score				
		CryoTrans -former	Topaz	crYOLO	GTpick		CryoTrans -former	Topaz	crYOLO	GTpick		CryoTrans -former	Topaz	crYOLO	GTpick
10081	39352	0.673	0.673	0.577	**0.681**		0.386	0.407	**0.506**	0.449		0.491	0.507	0.539	**0.541**
10093	56394	0.607	0.603	0.430	**0.609**		0.306	0.486	**0.527**	0.381		0.407	**0.538**	0.473	0.468
10532	87933	0.529	0.507	0.356	**0.535**		0.434	0.552	**0.673**	0.520		0.477	0.526	0.465	**0.527**
11056	125908	0.427	0.364	0.393	**0.440**		0.460	0.551	**0.576**	0.560		0.443	0.439	0.467	**0.493**
10345	15894	0.842	0.811	0.544	**0.850**		0.176	0.357	**0.525**	0.207		0.291	0.496	**0.535**	0.332
10947	106393	0.441	0.186	0.147	**0.460**		0.291	0.705	**0.750**	0.288		0.350	0.294	0.246	**0.354**
Average		0.587	0.524	0.408	**0.596**		0.342	0.510	**0.593**	0.401		0.410	**0.467**	0.454	0.453

Note: GTpick parameters: 62.34 M; CryoTransformer parameters: 75.94 M.

Across the six test datasets, GTpick consistently demonstrates superior recall performance, particularly achieving the highest values on challenging datasets such as EMPIAR-10081, EMPIAR-10532, EMPIAR-11056, and EMPIAR-10345, which feature complex backgrounds or densely distributed particles. Even on the large-scale EMPIAR-10947 dataset with a greater number of micrographs, GTpick maintains stable performance, highlighting its strong robustness and cross-dataset generalization capability.

As shown in Table 3, GTpick outperforms CryoTransformer, crYOLO, and Topaz in terms of recall, while also achieving competitive precision, resulting in overall higher F1-scores. In addition, GTpick has a model size of 62.34 million parameters, approximately 18 % fewer than CryoTransformer’s 75.94 million, demonstrating significantly reduced computational resource consumption without compromising detection accuracy.

The lower precision observed on the EMPIAR-10345 and EMPIAR-10947 datasets is mainly attributed to the intrinsic characteristics of these datasets.

Specifically, EMPIAR-10345 corresponds to a signaling protein complex, which features relatively large particle diameters (approximately 450 px) and a high particle density per micrograph. Such dense distributions often cause particle overlap or partial merging, increasing the likelihood of misclassification in boundary regions. This leads to a higher number of false positives and consequently reduces precision. In contrast, the EMPIAR-10947 dataset contains viral protein particles with smaller molecular weight (approximately 443 kDa) and lower image contrast. The low signal-to-noise ratio (SNR) and irregular particle morphology result in blurred particle boundaries, which tend to cause over-detection and thus lower precision. Nevertheless, GTpick maintains a relatively high recall on both datasets, indicating that the model remains highly sensitive to true particle regions.

**Fig. 4:**
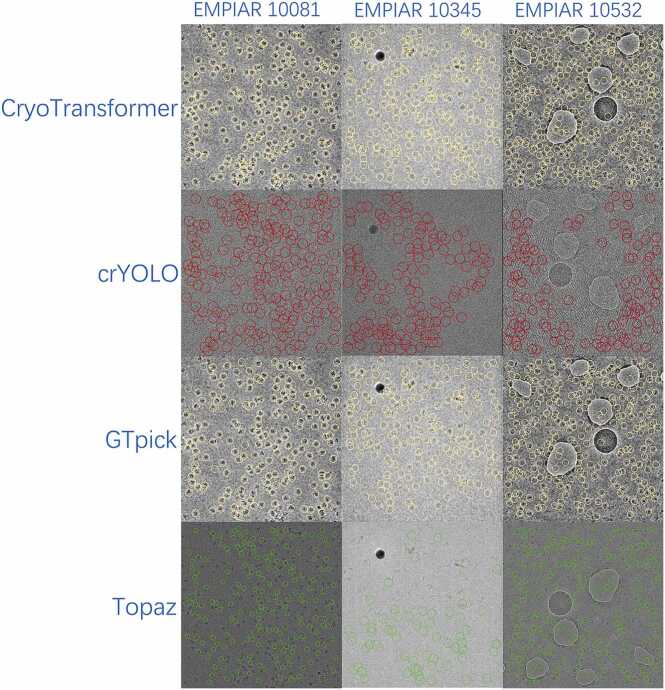
Visual comparison of particle detection results obtained by CryoTransformer, crYOLO, GTpick (ours), and Topaz on representative micrographs from EMPIAR-10081, EMPIAR-10345, and EMPIAR-10532.

To qualitatively assess the performance differences among particle detection algorithms, Fig. 4 presents the particle picking results of GTpick, CryoTransformer, crYOLO, and Topaz on representative micrographs from EMPIAR-10081, EMPIAR-10345, and EMPIAR-10532. Each colored overlay indicates the particle locations detected by the corresponding method: CryoTransformer (yellow), crYOLO (red), GTpick (yellow), and Topaz (green).

As shown in Fig. 4, CryoTransformer performs well on sparsely distributed particles but tends to merge adjacent particles or miss smaller ones in high-density regions. crYOLO exhibits high sensitivity but produces a large number of false positives, often misidentifying background noise as particles, particularly in EMPIAR-10532 with low contrast and variable particle size. Topaz, conversely, demonstrates a conservative detection behavior, frequently missing closely packed particles. In contrast, GTpick achieves a more accurate estimation of the true number of particles in each micrograph. This is particularly important for downstream 3D reconstruction, where the number and quality of picked particles directly determine the achievable resolution.

#### 4.2.2 Comparisons of 3D density map reconstruction and resolution

Next, we comprehensively evaluated GTpick’s performance in Cryo-EM particle detection from a 3D reconstruction perspective. To achieve this, five representative datasets—EMPIAR-10081, EMPIAR-10345, EMPIAR-10532, EMPIAR-10093, and EMPIAR-10947—were selected for systematic evaluation. The particles detected by each method were imported into the CryoSPARC platform for 3D density reconstruction. Reconstruction proceeded after 2D classification using the Select 2D module, which removed low-quality or abnormal particles to enhance the consistency of the input particle set and improve the accuracy of the final structure.

To ensure statistical reliability and experimental stability, three independent reconstruction experiments were conducted for each dataset and each particle detection method. All experiments followed identical preprocessing parameters and reconstruction workflows to maintain fairness and reproducibility.

**Fig. 5:**
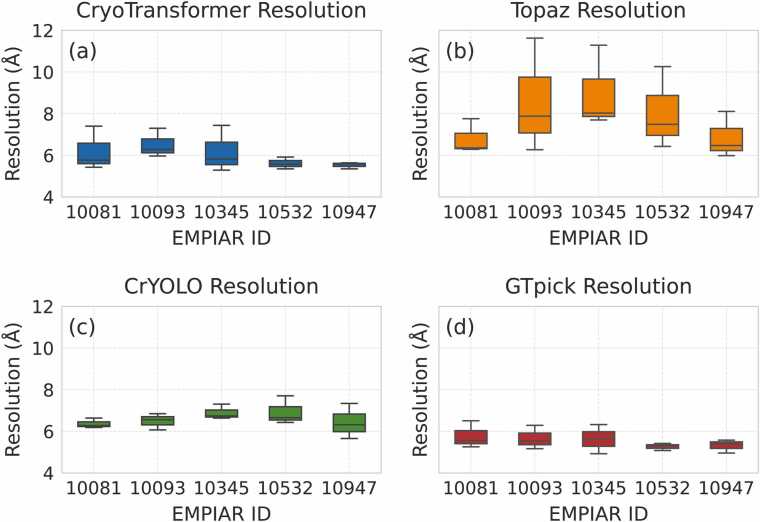
Boxplots of 3D reconstruction resolutions obtained from three independent experiments on four Cryo-EM datasets using different particle detection methods: (a) CryoTransformer, (b) Topaz, (c) crYOLO, and (d) GTpick. Lower resolution values (Å) indicate better reconstruction quality. GTpick exhibits smaller variance and lower median values, demonstrating higher stability and superior reconstruction performance.

The resolution distributions obtained from the three repeated experiments are illustrated in Fig. 5 as boxplots, enabling an intuitive comparison of reconstruction stability and overall performance among different methods. The smaller spread and lower median values observed in GTpick indicate its higher stability and accuracy in 3D reconstruction.

In addition to the boxplots, Table 4 summarizes the best reconstruction resolutions achieved across all datasets. Furthermore, the Fourier Shell Correlation (FSC) curves corresponding to these best reconstructions are provided in Fig. S1, further confirming the reliability and consistency of GTpick’s 3D reconstruction performance across different datasets. Meanwhile, Table S1 presents the complete resolution records from all three independent reconstruction experiments, offering a comprehensive quantitative reference. Overall, GTpick demonstrates higher reconstruction consistency and superior resolution performance across all datasets, showing a clear advantage over CryoTransformer, Topaz, and crYOLO.

**Table 4:** Comparison of 3D reconstruction resolution (Å) of different methods on the EMPIAR dataset.

EMPIAR ID	Number of Micrographs	3D Resolution (Å)			
		CryoTransformer	Topaz	crYOLO	GTpick
10081	300	7.30	7.76	6.56	**6.29**
10093	300	5.35	6.36	6.28	**5.26**
10345	300	5.82	10.26	6.66	**5.17**
10532	305	5.64	5.99	5.66	**5.41**
10947	400	5.29	7.49	7.31	**4.93**

The 3D reconstruction resolution was quantified using the Gold Standard Fourier Shell Correlation (GSFSC) criterion, which measures reconstruction accuracy and confidence. Lower GSFSC values correspond to higher fidelity and improved particle detection quality. As shown in Table 4, GTpick consistently achieved the best reconstruction resolution across all datasets. Specifically, GTpick reached 6.29 Å, 5.26 Å, 5.17 Å, 5.41 Å, and 4.93 Å on the EMPIAR-10081, EMPIAR-10093, EMPIAR-10345, EMPIAR-10532, and EMPIAR-10947 datasets, respectively—surpassing CryoTransformer, Topaz, and crYOLO. These results demonstrate that GTpick not only identifies a greater number of high-quality particles but also contributes to more accurate and higher-resolution 3D reconstructions.

**Fig. 6:**
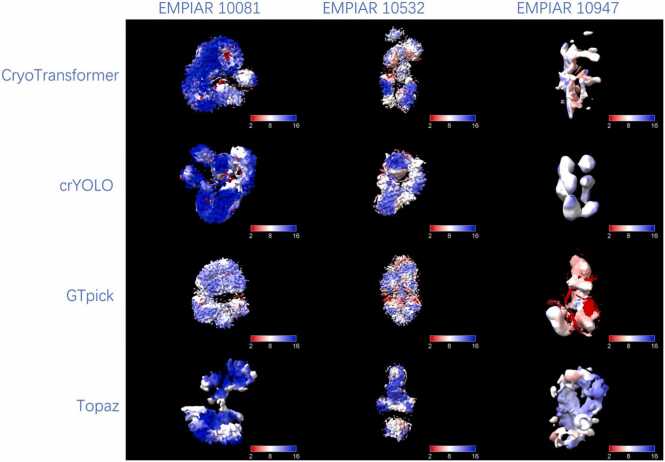
Local-resolution visualization of 3D reconstructions obtained from representative datasets using CryoTransformer, crYOLO, GTpick, and Topaz. Color bars indicate the local-resolution range from 2 Å (red, higher resolution) to 16 Å (blue, lower resolution). Compared with other particle-picking methods, GTpick yields more extensive high-resolution (red) regions and smoother density continuity, indicating improved structural fidelity and reconstruction consistency.

Furthermore, the reconstructed 3D density maps corresponding to the best resolutions were analyzed using UCSF ChimeraX to visualize the local resolution distribution of each map, as shown in Fig. 6. This visualization intuitively reveals the spatial variation of resolution within the reconstructed volumes and further demonstrates that GTpick yields more uniform and higher-quality 3D reconstructions compared with other particle picking methods.

To further evaluate the structural consistency between the reconstructed density maps and experimentally determined atomic models, the corresponding PDB structures were fitted into the density maps for visual comparison. Specifically, the fitted atomic models corresponding to EMPIAR-10081 and EMPIAR-10532 are 5U6O [31] and 6WXB [32], respectively. The overlay results, shown in Fig. S2, demonstrate clear structural alignment and spatial correspondence between the atomic models and the reconstructed maps. These visualizations further confirm that the reconstructions derived from GTpick-detected particles exhibit higher fidelity and better agreement with the known atomic structures.

Finally, Fig. S3 presents the complete local resolution maps for all five datasets, further validating GTpick’s capability and consistency in 3D reconstruction across different datasets.

### 4.3 Cross-dataset generalization evaluation and model generalization analysis

To further evaluate the cross-dataset generalization capability of GTpick, an additional experiment was conducted where the model was trained solely on the EMPIAR-10081 dataset and tested on five independent CryoPPP datasets, including EMPIAR-10093, EMPIAR-10532, EMPIAR-11056, EMPIAR-10345, and EMPIAR-10947. Unlike the main experiment that adopted an 8:1:1 train–validation–test split across multiple datasets, this test aimed to simulate a low-data and cross-domain scenario more representative of practical Cryo-EM applications, where annotated particle data are often scarce. In this setting, EMPIAR-10081 contained only 270 training and 30 validation micrographs. The limited data volume and lack of diversity reduced the model’s exposure to varying defocus levels, contrast patterns, and molecular orientations, leading to a clear performance decline when applied to unseen datasets.

As summarized in Table 5, GTpick exhibited varying levels of performance across the five test datasets, with Precision ranging from 0.108 to 0.335, Recall from 0.365 to 0.693, and F1-scores between 0.187 and 0.383.

**Table 5:** Performance metrics of the GTpick model trained on the EMPIAR-10081 dataset and evaluated on five independent CryoPPP datasets (EMPIAR-11056, EMPIAR-10093, EMPIAR-10345, EMPIAR-10532, and EMPIAR-10947) for cross-dataset generalization assessment.

EMPIAR ID	Precision	Recall	F1 Score
10093	0.209	0.483	0.292
10532	0.316	0.485	0.383
11056	0.335	0.365	0.349
10345	0.108	0.693	0.187
10947	0.243	0.368	0.293

These fluctuations highlight the inherent sensitivity of deep learning–based particle pickers to dataset characteristics such as noise distribution, particle density, and imaging contrast.

Nevertheless, GTpick retained reasonable recall performance (e.g., 0.483 on EMPIAR-10093 and 0.693 on EMPIAR-10345), demonstrating its ability to identify potential particle regions even under domain shifts.

## 5 Discussion

In this study, we present GTpick, a novel detection method based on the DETR framework, designed to address the core challenges of low signal-to-noise ratios (SNRs) and densely distributed targets in cryo-electron microscopy (Cryo-EM) particle detection. This method significantly enhances target feature modeling and contextual representation while maintaining a compact network architecture and low training overhead, thereby improving detection accuracy and robustness. When integrated with the CryoSPARC 3D reconstruction workflow, GTpick produces higher-resolution density maps, leading to more accurate and reliable protein structure determination.

Evaluation on multiple EMPIAR datasets demonstrates that GTpick outperforms existing methods such as CryoTransformer and Topaz, achieving superior particle recognition and improved 3D reconstruction quality across multiple test sets. These findings further emphasize the critical role of high-quality particle detection in enabling accurate structural reconstruction.

Despite GTpick’s strong performance in particle count, recall rate, and reconstruction quality, the broader target coverage is accompanied by a certain level of false detections. Future research could focus on optimizing target matching mechanisms and post-processing strategies to further reduce false positives and improve precision across diverse datasets.

Moreover, an interesting and open question remains: human experts still outperform machine-learning-based methods in particle selection accuracy. This phenomenon highlights the sophisticated cognitive integration of spatial, contextual, and contrast cues that current algorithms cannot yet fully emulate. Nevertheless, we also emphasize the promising potential of emerging deep-learning paradigms, such as self-supervised learning, multimodal data integration, and domain adaptation, which may enable future methods to reach or even surpass human-level performance.

In such a scenario, reliance on human-labeled datasets as the sole ground truth will become increasingly limited, motivating the development of new evaluation frameworks based on structural consistency and density-map fidelity, thereby providing a more objective and comprehensive assessment of automated particle detection and structural reconstruction quality.

## Code and data availability

To support reproducibility and further research, the full GTpick training and evaluation pipeline is available at: https://github.com/nshstart/GTpickcom3.git. The datasets used in this study are publicly available.

## CRediT authorship contribution statement

**Shenhuan Ni:** Writing – review & editing, Methodology, Data curation, Conceptualization. **Anthony Qian:** Writing – review & editing. **Ren Kong:** Supervision, Conceptualization. **Shan Chang:** Writing – review & editing, Supervision, Methodology, Conceptualization. **Chenghui Yang:** Writing – review & editing, Conceptualization. **Yutao Liu:** Writing – review & editing, Conceptualization. **Yuncong Zhang:** Writing – review & editing, Conceptualization. **Yiyan Shi:** Writing – review & editing.

## Funding

This work was financially supported by the funding received from the 10.13039/501100001809National Natural Science Foundation of China (Nos. 62373172).

## Declaration of Competing Interest

The authors declare that there are no conflicts of interest regarding the publication of this paper.

## Footnotes

Appendix A Supplementary data associated with this article can be found in the online version at doi:10.1016/j.csbj.2025.10.029.
