# Supplementary material for: GTpick: A deep neural network for Cryo-EM particle detection
Source: Comput Struct Biotechnol J. 2025 Oct 16;27:4451–8. doi: 10.1016/j.csbj.2025.10.029 (PMC12590290; doi:10.1016/j.csbj.2025.10.029)
Supplement: Supplementary file 1 — Supplementary material [file mmc1.docx]

**Table S1.** Resolutions (Å) obtained from three independent 3D reconstruction trials on five representative Cryo-EM datasets (EMPIAR-10081, EMPIAR-10093, EMPIAR-10345, EMPIAR-10532, and EMPIAR-10947) using four particle detection methods: CryoTransformer, Topaz, crYOLO, and GTpick.

| EMPIAR ID | CryoTransformer | | | Topaz | | | crYOLO | | | GTpick | | |
| --- | --- | --- | --- | --- | --- | --- | --- | --- | --- | --- | --- | --- |
|  | Resolution for 3 Trials (Å) | | | Resolution for 3 Trials (Å) | | | Resolution for 3 Trials (Å) | | | Resolution for 3 Trials (Å) | | |
|  | 1 | 2 | 3 | 1 | 2 | 3 | 1 | 2 | 3 | 1 | 2 | 3 |
| 10081 | 7.40 | 7.30 | 7.44 | 7.76 | 7.88 | 8.03 | 6.64 | 6.56 | 6.64 | 6.51 | 6.29 | 6.33 |
| 10093 | 5.35 | 5.35 | 5.43 | 6.43 | 6.47 | 6.36 | 6.43 | 6.32 | 6.28 | 5.30 | 5.58 | 5.26 |
| 10345 | 6.28 | 5.82 | 5.92 | 11.63 | 11.29 | 10.26 | 6.85 | 6.74 | 6.66 | 5.17 | 5.64 | 5.42 |
| 10532 | 5.64 | 5.77 | 5.97 | 5.99 | 6.29 | 6.27 | 5.66 | 6.19 | 6.07 | 5.41 | 5.56 | 5.55 |
| 10947 | 5.29 | 5.58 | 5.59 | 7.70 | 7.49 | 8.11 | 7.31 | 7.71 | 7.34 | 4.93 | 5.08 | 4.96 |


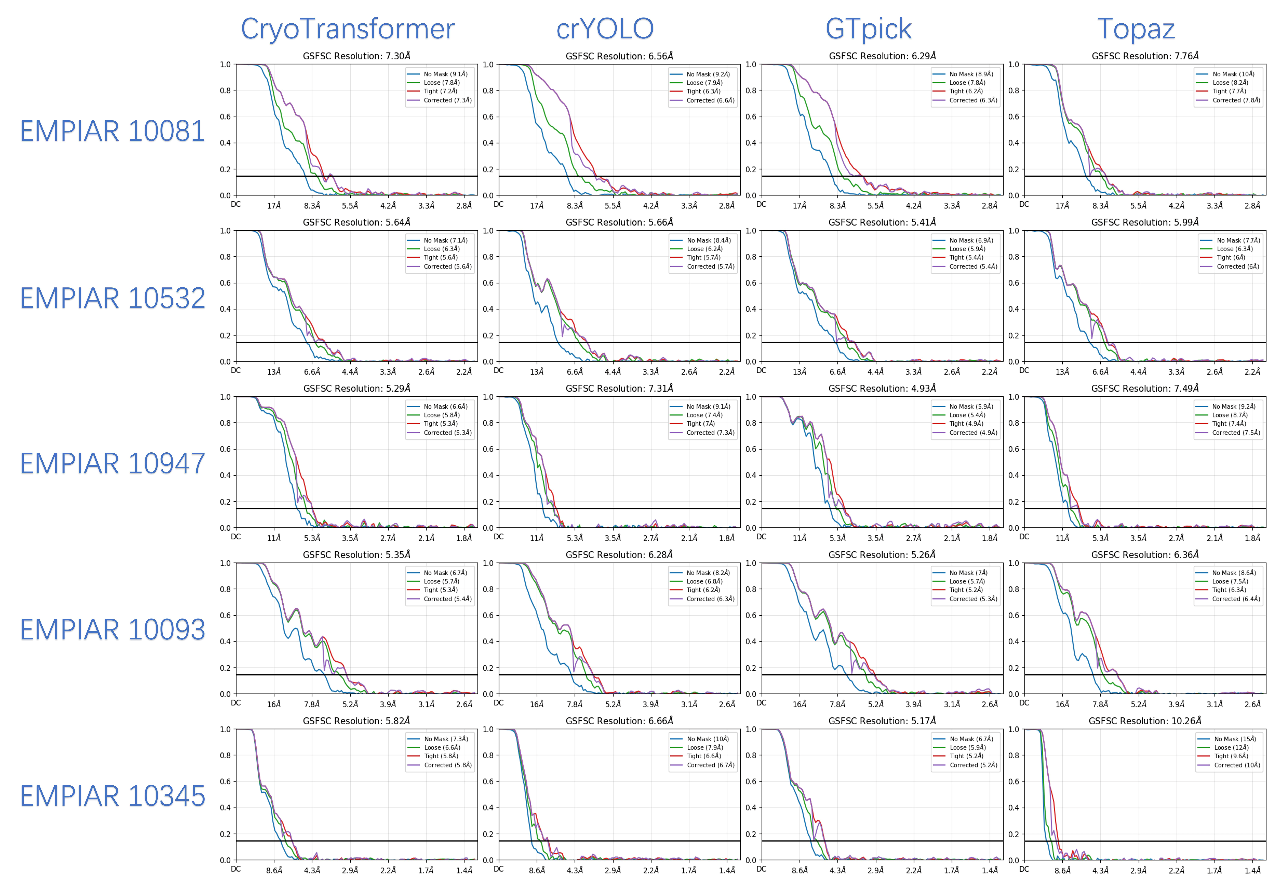


**Fig. S1.** Fourier Shell Correlation (FSC) curves of the best 3D reconstructions obtained using CryoTransformer, crYOLO, GTpick, and Topaz across five EMPIAR datasets (10081, 10532, 10345, 10093, 10947). All FSC curves were calculated using the Gold Standard FSC (GSFSC) criterion in CryoSPARC. GTpick shows higher FSC values across frequencies, indicating improved reconstruction accuracy. The 0.143 line denotes the resolution cutoff.


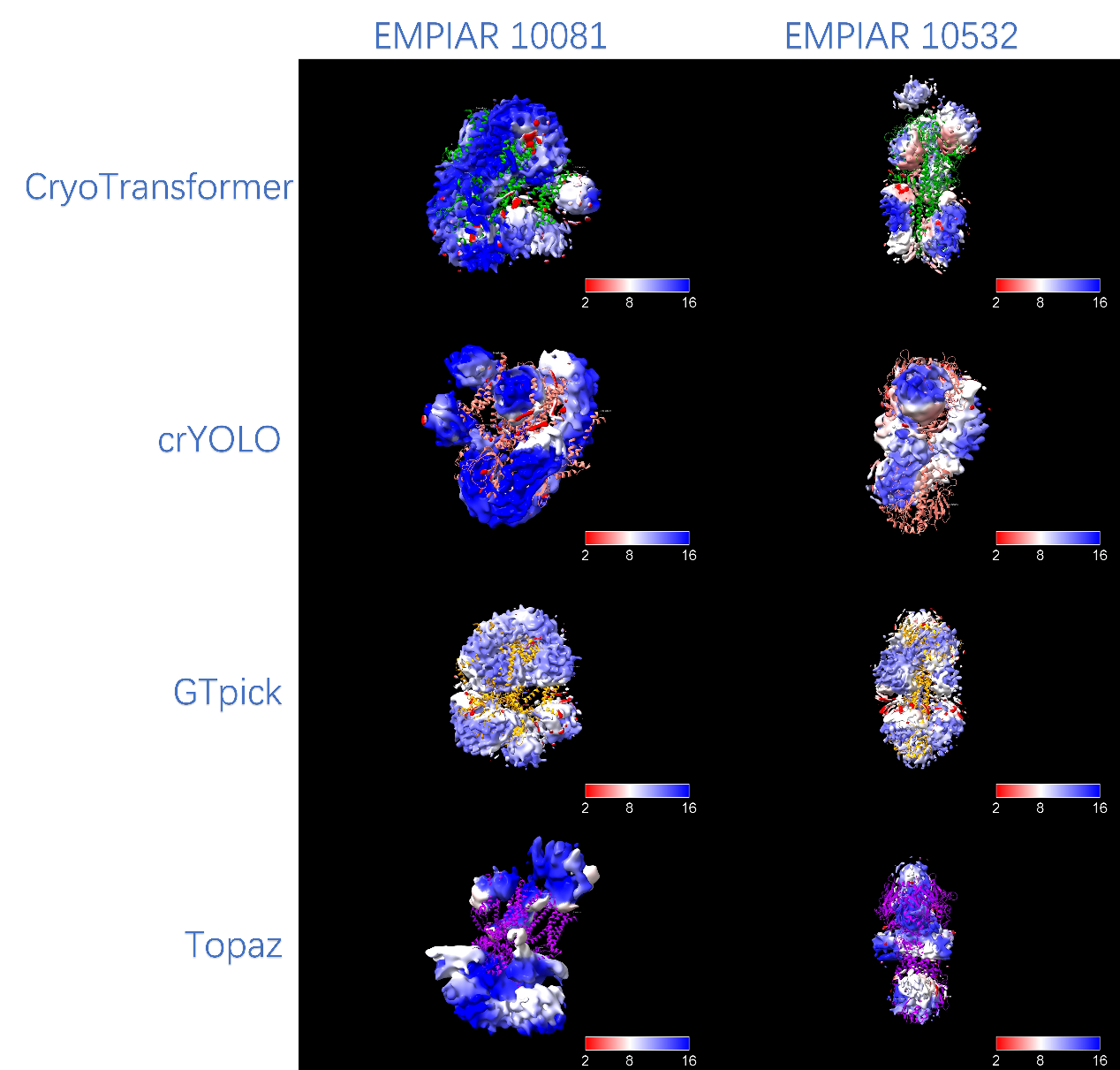


**Fig. S2.** Comparison of reconstructed 3D density maps and corresponding fitted atomic models for EMPIAR-10081 and EMPIAR-10532 datasets using CryoTransformer, crYOLO, GTpick (ours), and Topaz. The local resolution distributions were visualized in UCSF ChimeraX, with color bars ranging from 2 Å (red, high resolution) to 16 Å (blue, low resolution). For structural validation, the fitted atomic models corresponding to EMPIAR-10081 and EMPIAR-10532 are PDB IDs 5U6O[1] and 6WXB[2], respectively. The atomic models are rendered in green (CryoTransformer), pink (crYOLO), yellow (GTpick), and purple (Topaz).


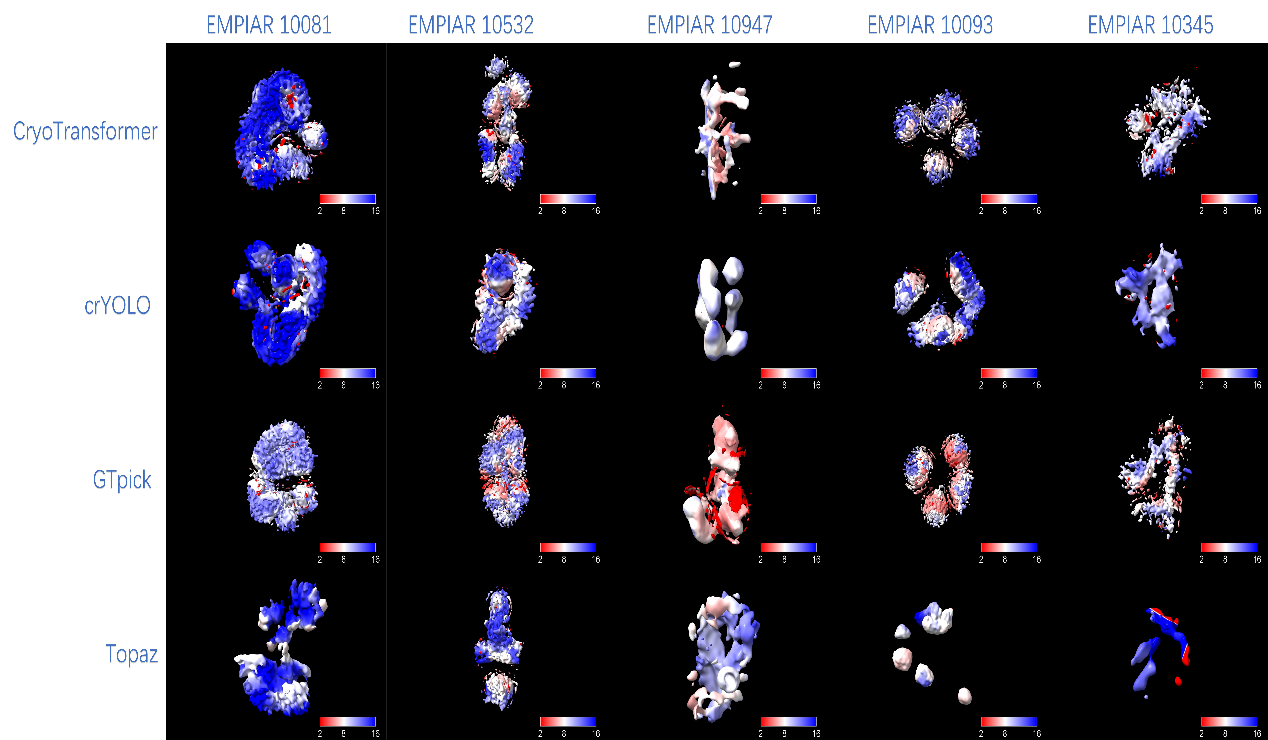


**Fig. S3.** Complete visualization of local-resolution maps for all five Cryo-EM datasets (EMPIAR-10081, EMPIAR-10532, EMPIAR-10345, EMPIAR-10093, and EMPIAR-10947) reconstructed using CryoTransformer, crYOLO, GTpick (ours), and Topaz. The local-resolution distributions were generated in UCSF ChimeraX, with color bars indicating the resolution range from 2 Å (red, high resolution) to 16 Å (blue, low resolution).

## **References**

1. Lee C-H, MacKinnon RJC. Structures of the human HCN1 hyperpolarization-activated channel. 2017;168(1):111-20. e11.

2. Tan YZ, Rubinstein JLJBC. Through-grid wicking enables high-speed cryoEM specimen preparation. 2020;76(11):1092-103.
